# Genomic and SNP Analyses Demonstrate a Distant Separation of the Hospital and Community-Associated Clades of *Enterococcus faecium*

**DOI:** 10.1371/journal.pone.0030187

**Published:** 2012-01-26

**Authors:** Jessica Galloway-Peña, Jung Hyeob Roh, Mauricio Latorre, Xiang Qin, Barbara E. Murray

**Affiliations:** 1 Division of Infectious Disease, Department of Medicine, University of Texas Medical School, Houston, Texas, United States of America; 2 Center for the Study of Emerging and Reemerging Pathogens, University of Texas Medical School, Houston, Texas, United States of America; 3 Department of Microbiology and Molecular Genetics, University of Texas Medical School, Houston, Texas, United States of America; 4 Laboratorio de Bioinformática y Expresión Génica, INTA, Universidad de Chile, Santiago, Chile; 5 Human Genome Sequencing Center, Baylor College of Medicine, Houston, Texas, United States of America; University of Nottingham, United Kingdom

## Abstract

Recent studies have pointed to the existence of two subpopulations of *Enterococcus faecium*, one containing primarily commensal/community-associated (CA) strains and one that contains most clinical or hospital-associated (HA) strains, including those classified by multi-locus sequence typing (MLST) as belonging to the CC17 group. The HA subpopulation more frequently has IS16, pathogenicity island(s), and plasmids or genes associated with antibiotic resistance, colonization, and/or virulence. Supporting the two clades concept, we previously found a 3–10% difference between four genes from HA-clade strains vs. CA-clade strains, including 5% difference between *pbp5-R* of ampicillin-resistant, HA strains and *pbp5-S* of ampicillin-sensitive, CA strains. To further investigate the core genome of these subpopulations, we studied 100 genes from 21 *E. faecium* genome sequences; our analyses of concatenated sequences, SNPs, and individual genes all identified two distinct groups. With the concatenated sequence, HA-clade strains differed by 0–1% from one another while CA clade strains differed from each other by 0–1.1%, with 3.5–4.2% difference between the two clades. While many strains had a few genes that grouped in one clade with most of their genes in the other clade, one strain had 28% of its genes in the CA clade and 72% in the HA clade, consistent with the predicted role of recombination in the evolution of *E. faecium*. Using estimates for *Escherichia coli*, molecular clock calculations using sSNP analysis indicate that these two clades may have diverged ≥1 million years ago or, using the higher mutation rate for *Bacillus anthracis*, ∼300,000 years ago. These data confirm the existence of two clades of *E. faecium* and show that the differences between the HA and CA clades occur at the core genomic level and long preceded the modern antibiotic era.

## Introduction

Over the past 30 years, the epidemiology of enterococcal infections has changed with *Enterococcus faecium* progressively increasing from causing ∼5% of enterococcal infections to now causing ∼35% of these infections [1], [2]. Several studies have shown that *E. faecium* strains that cause hospital-associated infections are often different from strains that colonize the gastro-intestinal tracts of community-based healthy individuals and food animals, with the former having higher frequencies of ampicillin resistance, *esp_efm_*, *hyl_efm_*, microbial surface components recognizing adhesive matrix molecules (MSCRAMMs), and the presence of IS16 [1], [3], [4], [5], [6], [7], [8], [9], [10], [11]. Early population-based studies by Willems and colleagues using multi-locus sequence typing (MLST) and the algorithm eBURST suggested that strains from nosocomial infections belonged to a distinct genetic lineage named Clonal Complex 17 [5], [12], [13]. It has since been reported using Bayesian modeling and other methods that eBURST-based clustering is inaccurate for determining evolutionary decent for species, like *E. faecium*, with high levels of recombination [14], [15], [16], [17]. Nonetheless, other studies based on comparative genome array, amplified fragment length polymorphism (AFLP), and pyrosequencing have also indicated the existence of two different subpopulations or “clades” in which the clinical, hospital-associated (HA) strains belong to a group that is distinct from the group that consists primarily of non-clinical, community-associated (CA) strains, such as those found in the stools of healthy individuals in the community [6], [18].

Some of the aforementioned publications have suggested that the presence of specific accessory genes and IS elements in HA strains has been the driving force behind the evolution of this organism [6], [18]. It has been hypothesized that these elements contributed to the ecological success of *E. faecium* and that acquiring adaptive mechanisms under selective pressure was pivotal for survival and proliferation in the hospital environment and/or in patients [6], [18]. That is, the acquisition of genes, rather than evolutionary descent, was predicted to be the driving force in determining the fitness of *E. faecium* strains [6], [18]. Although the acquisition of antibiotic resistance and virulence/fitness determinants has almost certainly contributed to the persistence and success of some members of this species in the nosocomial environment, there may be fundamental differences between the two subpopulations at the level of the core genome that have also contributed to the success of hospital-associated strains. Fundamental differences have been suggested by analysis of pyrosequenced genomes [18] and by our previous study's findings that strains separated clearly into two distinct groups based on four genes, with a 3% nucleotide difference for *gls20*, 5% for *pbp5*, 7% for *pbp2*, and 10% for *wlcA* between these genes from strains in the two groups [19].

In this study, genomic, SNP, and phylogenetic analyses showed that the differences between HA-clade strains and CA-clade strains are found throughout the core genome. In addition, molecular clock estimations indicate that the divergence of these two clades was a distant evolutionary event.

## Results

### Analysis of 100 genes from 21 *E. faecium* genomes illustrates the differences in core genome components of two clades

**Figure 1:**
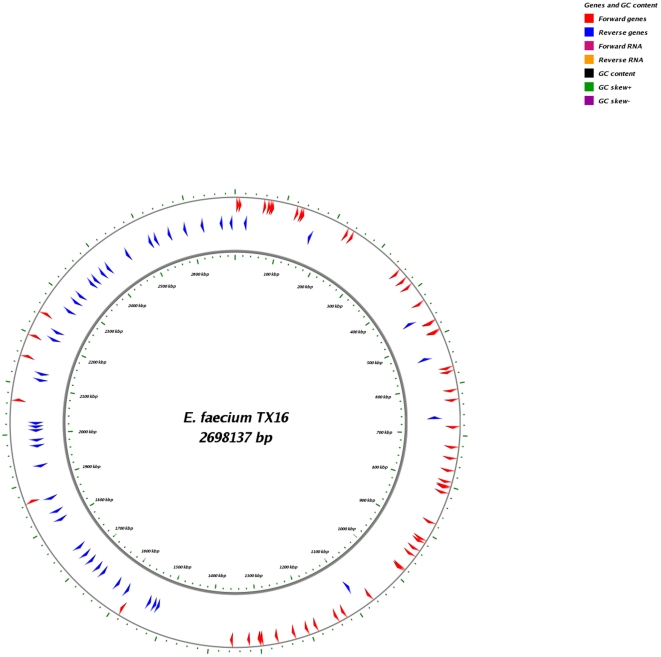
Position of the 100 genes analyzed on the TX16 (DO) chromosome. The chromosomal map of the *Enterococcus faecium* genome of the endocarditis isolate TX16, commonly referred to as DO. The arrows represent the 100 genes chosen for clade and SNP analysis and their position on the chromosome. The red arrows depict genes transcribed in the forward orientations, and blue arrows depict genes transcribed in the reverse orientation. The general location of a few subset of the 100 genes are labeled.

A total of 1608 orthologs (defined as >80% identity and aligning to >60% match length) were common to all 21 genomes; 638 of these had the same DNA length in the available sequences from all the genomes analyzed. The 100 gene sequences chosen (Figure 1 and Table S1) had ≥95% identity and were concatenated into a 106,818 bp nucleotide sequence for each of the strains (information on genomes listed in Table 1). The comparisons between all the strains were determined and are shown in Table 2. The nucleotide percent identities among the strains within the CA clade (in yellow) ranged from 98.9%–100%, and the divergence scores from 0–1.2; the nucleotide percent identities between the strains in the HA clade (in green) range from 99–100% and the divergence scores from 0–1 (Table 2). However, when the two clades were compared to each other, the percent identities ranged from 95.8–96.5% and the divergence scores from 3.6–4.3 (Table 2). Therefore, 1141733, Com12, Com15, E980, and TX1330 are more closely related to each other than to the other 16 *E. faecium* strains and in turn, these 16 strains were found to be more related to each other than to the above 5 strains, reinforcing the grouping into two distinct clades.

**Table 1:** The sequence type, country, date, and source of isolation for the 21 sequenced *E. faecium* genomes used in this study.

Strain	ST^a^	Country^b^	year	Source	Accession #
C68	16	USA (OH)	1998	Endocarditis patient (blood)	ACJQ00000000
Com12	107	USA	2006	Healthy volunteer fecal sample	ACBC00000000
Com15	583	USA	2006	Healthy volunteer fecal sample	ACBD00000000
D344SRF	21	France	1985	Clinical isolate	ACZZ00000000
E980	94	Netherlands	1998	Healthy volunteer fecal sample	ABQA01000001
E1039	42	Netherlands	1998	Healthy volunteer fecal sample	ACOS00000000
E1071	32	Netherlands	2000	Hospitalized patient fecal sample	ABQI01000001
E1162	17	France	1997	Blood Culture of Hospitalized Patient	ABQJ00000000
E1636	106	Netherlands	1961	Blood Culture of Hospitalized Patient	ABRY01000001
E1679	114	Brazil	1998	Swab of Vascular Catheter Tip	ABSC01000001
TX16	18	USA (TX)	1992	Endocarditis patient (blood)	ACIY00000000
TX82	17	USA (TX)	1999	Endocarditis patient (blood)	AEBU00000000
TX0133	17	USA (TX)	2006	Endocarditis patient (blood)	AECH00000000
TX1330	107	USA	1994	Healthy volunteer fecal sample	ACHL00000000
U0317	78	Netherlands	2005	UTI of Hospitalized Patient (Urine)	ABSW01000001
1141733	327	unk.	unk.	Blood Culture of Hospitalized Patient	ACAZ00000000
1230933	18	unk.	unk.	Wound Swab of Hospitalized Patient	ACAS00000000
1231408	582	unk.	unk.	Blood Culture of Hospitalized Patient	ACBB00000000
1231410	17	unk.	unk.	Skin and Soft Tissue Infection	ACBA00000000
1231501	52	unk.	unk.	Blood Culture of Hospitalized Patient	ACAY00000000
1231502	203	unk.	unk.	Blood Culture of Hospitalized Patient	ACAX00000000

a ST is the sequence type by multilocus sequence typing.

b “unk.” means the information for this isolate is unknown.

**Table 2:** Percent identity and divergence score matrix of the 100 concatenated gene nucleotide sequence.

Percent Identity (%)																						
Divergence		1141733^a^	Com12	Com15	E980	TX1330	1231408^b^	1230933^c^	1231410	1231501	1231502	C68	D344SRF	TX16	E1039	E1071	E1162	E1636	E1679	TX82	TX0133A	U0317
	**1141733**		*99.8*	*98.9*	*98.8*	*99.8*	**96.8**	95.9	96	96.2	96	95.8	95.9	95.9	96.1	96	95.9	96	95.9	95.9	95.8	96
	**Com12**	*0.2*		*98.9*	*98.9*	*100*	**96.7**	96	96	96.2	96	95.8	95.9	96	96.2	96	95.9	96	96	95.9	95.9	96
	**Com15**	*1.1*	*1.1*		*99.2*	*98.9*	**96.6**	95.9	96	96.2	96.1	95.8	95.9	95.9	96.2	96.1	96	96.1	96	96	95.9	96.1
	**E980**	*1.2*	*1.1*	*0.8*		*98.9*	**96.6**	96.2	96.3	96.5	96.3	96	96.2	96.2	96.5	96.3	96.2	96.3	96.2	96.2	96.2	96.3
	**TX1330**	*0.2*	*0*	*1.1*	*1.1*		**96.7**	96	96	96.2	96	95.8	95.9	96	96.2	96	95.9	96	96	95.9	95.9	96
	**1231408**	**3.3**	**3.4**	**3.5**	**3.5**	**3.4**		**98.9**	**98.8**	**98.4**	**98.9**	**98.7**	**98.8**	**98.9**	**98.5**	**98.8**	**98.9**	**98.5**	**98.7**	**98.9**	**98.9**	**98.9**
	**1230933**	4.2	4.2	4.2	3.9	4.2	**1.1**		99.9	99.2	99.8	99.6	99.7	100	99.2	99.6	99.8	99.4	99.6	99.8	99.8	99.8
	**1231410**	4.1	4.1	4.1	3.9	4.1	**1.2**	0.1		99.2	99.8	99.7	99.6	99.9	99.2	99.6	99.9	99.3	99.5	99.9	99.8	99.8
	**1231501**	3.9	3.9	3.9	3.6	3.9	**1.6**	0.8	0.9		99.1	99	99.3	99.2	99.2	99.1	99.2	99.4	99.2	99.2	99.3	99.1
	**1231502**	4.1	4.1	4.1	3.8	4.1	**1.1**	0.2	0.2	0.9		99.5	99.5	99.8	99.2	99.7	99.7	99.2	99.5	99.7	99.7	100
	**C68**	4.4	4.4	4.4	4.1	4.4	**1.3**	0.4	0.3	1	0.5		99.5	99.6	99	99.5	99.8	99.2	99.4	99.8	99.7	99.5
	**D344SRF**	4.3	4.2	4.2	3.9	4.2	**1.2**	0.3	0.4	0.7	0.5	0.5		99.7	99.2	99.5	99.7	99.7	99.7	99.7	99.8	99.5
	**TX16**	4.2	4.2	4.2	3.9	4.2	**1.1**	0	0.1	0.8	0.2	0.4	0.3		99.2	99.6	99.8	99.4	99.5	99.8	99.8	99.8
	**E1039**	4	4	3.9	3.7	4	**1.5**	0.8	0.8	0.8	0.8	1	0.8	0.8		99.2	99.2	99.5	99.2	99.2	99.2	99.2
	**E1071**	4.1	4.1	4.1	3.8	4.1	**1.2**	0.4	0.4	0.9	0.3	0.5	0.5	0.4	0.8		99.7	99.3	99.6	99.7	99.6	99.7
	**E1162**	4.2	4.2	4.2	3.9	4.2	**1.1**	0.2	0.1	0.8	0.3	0.2	0.3	0.2	0.8	0.3		99.4	99.6	100	99.9	99.7
	**E1636**	4.1	4.1	4.1	3.8	4.1	**1.5**	0.6	0.7	0.6	0.8	0.8	0.3	0.6	0.5	0.7	0.6		99.5	99.4	99.5	99.2
	**E1679**	4.2	4.2	4.2	3.9	4.2	**1.3**	0.4	0.5	0.8	0.5	0.6	0.3	0.5	0.8	0.4	0.4	0.5		99.6	99.7	99.5
	**TX82**	4.2	4.2	4.2	3.9	4.2	**1.1**	0.2	0.1	0.8	0.3	0.2	0.3	0.2	0.8	0.3	0	0.6	0.4		99.9	99.7
	**TX0133A**	4.3	4.3	4.3	4	4.3	**1.1**	0.2	0.2	0.7	0.3	0.3	0.2	0.2	0.8	0.4	0.1	0.5	0.3	0.1		99.7
	**U0317**	4.1	4.1	4.1	3.8	4.1	**1.2**	0.2	0.2	0.9	0	0.5	0.5	0.2	0.8	0.3	0.3	0.8	0.5	0.3	0.3	

a The numbers in italics (upper left) are the percent identity and divergence scores of the CA strains.

b The numbers in bold are the percent identity and divergence scores of the hybrid strain.

c The numbers in regular text (down right) are the percent identity and divergence scores of the HA strains.

**Figure 2:**
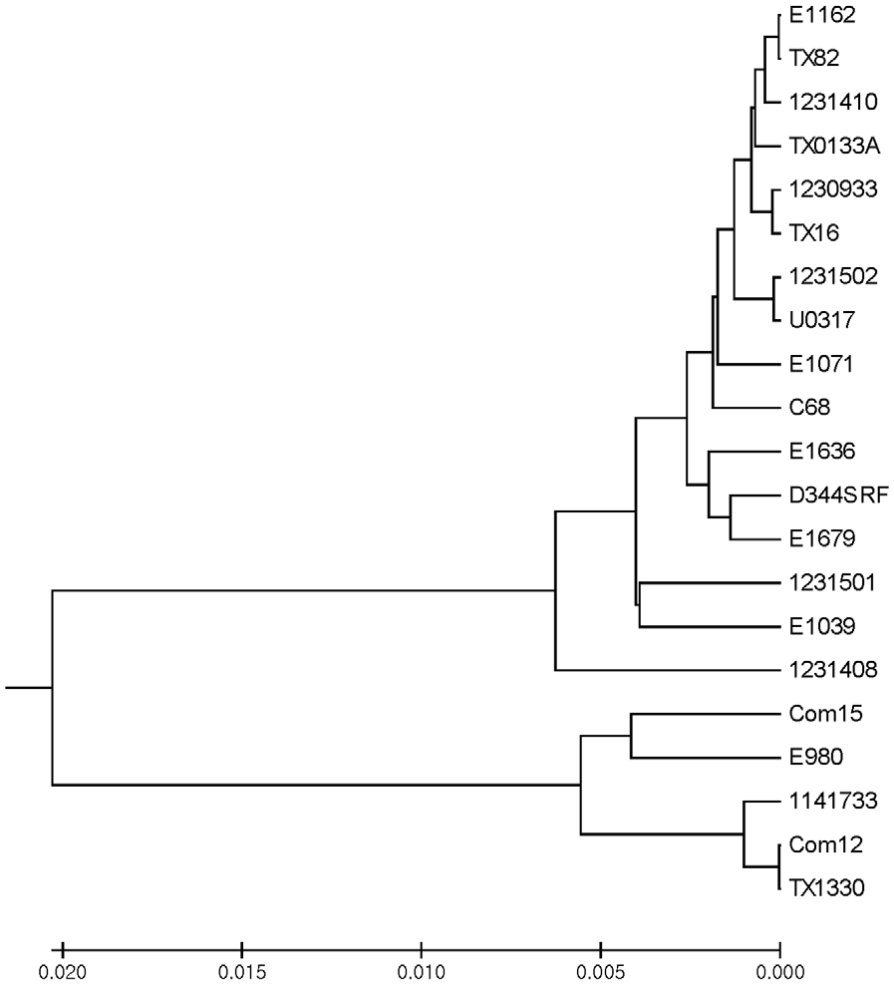
UPGMA phylogenetic tree of the concatenated 100 gene sequence (106,818 bp). The results are based on a pair-wise analysis of the concatenated 100 gene nucleotide sequence for each of the 21 *E. faecium* strains using the Poisson correction method in MEGA4.0.2 software. An UPGMA tree was constructed depicting the evolutionary distance between these sequences. The tree is drawn to scale with the branch lengths representing the evolutionary distances, the scale can be seen at the bottom of the tree.

The phylogenetic tree constructed using the concatenated sequences is shown in Figure 2. The tree separates the genomes into two distinct groups with one group consisting primarily of the strains from healthy volunteer fecal samples (Com12, Com15, TX1330, and E980), while most of the strains from clinical samples were in the other branch. Similar to previous terminology [6], [18], we designated these two branches the community-associated (CA) clade and the hospital-associated (HA) clade, respectively. There were, however, two exceptions. Strain 1141733 (which is a blood culture from a hospitalized patient) grouped in the branch with the commensal, fecal strains, and E1039, which is a healthy volunteer fecal sample, grouped with the clinical strains.

One *E. faecium* strain, 1231408 (in blue), isolated from the blood of a hospitalized patient, was found to have a “hybrid” genome sequence of both groups. The sequence between ORF No. 10017 and 10683 of 1231408 was found to be more similar to the sequences of strains in the CA clade whereas the rest of the 1231408 sequence was more similar to *E. faecium* strains in the HA clade. The overall percent identities of 1231408 ranged from 98.4–98.9% against HA clade strains and 96.7–96.8% against CA clade strains with divergence scores ranging from 1.1–1.6 against HA clade strains, and 3.3–3.5 against CA clade strains (Table 2).

The sequences of the individual genes from each of the 21 genomes were also compared and UPGMA trees constructed (data not shown) to create 100 individual trees; 92 of the 100 genes split the genomes into two distinct groups, while the other 8 genes were either 100% conserved or the sequences were not distinct enough to give two clear groupings (Table S1). For each genome, we counted the genes that segmented into the HA clade or the CA clade (Table 3). The CA clade strains ranged from strain 1141733 with 92 of 92 genes in the CA clade to E980 with 84/92 genes in the CA clade and 8/92 genes within the HA clade. The HA clade strains ranged from D344SRF, TX82, TX0133, E1162, and C68, all with 92/92 genes grouping with the HA clade strains, to E1039 with 86/92 genes grouping with the HA clade and 6/92 genes in the CA clade. The hybrid strain 1231408 had 66/92 genes group with the HA clade and the other 26 with the CA clade, consistent with the percent identity and divergence scores above.

**Table 3:** Analysis of 92 individual genes.

Community Clade	Hospital Clade	Hybrid Clade
1141733 (92+0)^a^	C68 (0+92)	1231408 (26+66) 66)
Com12 (91+1)	D344SRF (0+92)	
TX1330 (91+1)	E1162 (0+92)	
Com15 (90+2)	TX82 (0+92)	
E980 (84+8)	TX0133 (0+92)	
	TX16 (1+91)	
	1230933 (1+91)	
	1231410 (1+91)	
	E1071 (2+90)	
	E1636 (2+90)	
	E1679 (4+88)	
	U0317 (4+88)	
	1231502 (4+88)	
	E1039 (5+87)	
	1231501 (5+87)	

a Numbers in parentheses are given as number of genes which grouped with the community-associated clade plus the number of genes that grouped with the hospital-associated clade.

The encoded proteins of the 100 genes were also concatenated, resulting in a 35,606 amino acid sequence. The percent identities and divergence scores were tabulated as well as a UPGMA phylogenetic tree constructed for the concatenated protein sequence (Table S2 and Figure S1, respectively). As expected, the results were similar to the nucleotide results, grouping the strains into two distinct clades.

### SNP analysis emphasizes clade-specific differences

5,932 SNP sites (5.6% of the total sequence) were identified in the 106,818 bp aligned sequences. Among these, 929 SNPs were strain specific SNPs (identified in only one strain) and the remaining 5,003 SNPs were identified in two or more *E. faecium* strains. Each SNP was concatenated into a continuous sequence; the percent identities and divergent scores for this analysis can be seen in Table S3 and the UPGMA tree seen in Figure S2. The percent identities of the concatenated SNP sequences among the CA clade strains ranged from 79–100% and the divergence scores from 0–26. Within the HA clade, the percent identities of the SNP sequences ranged from 82–100% and the divergence scores from 0–21. When the two clades were compared by pair-wise analysis of each strain to one another, the percent identities ranged from 24–37% and the divergence score was 350. For the hybrid strain 1231408, the identities ranged from 38–42% and the divergence scores 23–39 with CA clade strains and 71–81% and 350 with HA clade strains.

Of the 5,932 SNPs, 5,147 (86.8%) were synonymous SNPs, whereas 785 (13.2%) were non-synonymous and found in 87 out of the 100 genes. Using the criteria described in the methods, 479 of the 785 non-synonymous SNPs were considered clade specific and were found in 72 of the 100 genes (Table S1). When searching through the KEGG map, the clade specific SNPs were found in 33 different metabolic pathways and 5 different genetic/environmental/cellular processes (Table S1).

### Molecular clock determination using sSNP analysis shows an evolutionarily distant separation between the CA and HA clades

We estimated the time of divergence using sSNP [20], [21], [22], [23]. The median number of sSNP in the CA clade was 979 (ranging from 1–1077), the median number of sSNPs in the HA clade was 401 (ranging from 2–916), and the median number of sSNPs between the two clades was 3812 (ranging from 3368–4065). These numbers were used to calculate the molecular clock and the results are shown in Table 4. Using the *E. coli* synonymous mutation rate and a range of generation times of 100–300 per year, the molecular clock determination estimated that strains within the CA clade diverged from each other on the average of ∼300,000 to 900,000 years ago, whereas strains within the HA clade diverged on the average of ∼100,000–300,000 years ago. When we used the same formula to calculate the divergence between the two clades, it is estimated that the two clades diverged between one million and three million years ago. Even with the faster mutational rate previously used with *B. anthracis*, the time of divergence was estimated at 300,000 years between the two clades (Table 4).

**Table 4:** Molecular clock/time of divergence estimates based on sSNP analysis within and between clades.

Based on the mutation rate of *Escherichia coli* (1.4×10^−10^)			
Generations/yr	Hospital Clade	Community Clade	Two clades
100	380,535	929,036	3,617,453
200	190,267	464,518	1,808,726
300	126,845	309,679	1,205,818

Based on the mutation rate of *Bacillus anthracis* (5.2×10^−10^)			
Generations/yr	Hospital Clade	Community Clade	Two clades
100	102,452	250,125	973,930
200	51,226	125,063	486,965
300	34,151	83,375	324,643

### 16S rRNA and ribosomal protein analyses show clade-specific SNPs and a similar time of divergence to the SNP analysis

One specific SNP at base-pair 61 out of 1569 differed between the strains following the CA and HA clade grouping as seen in the other analyses (Figure S3A). CA clade strains had a thymine at this position, while HA clade strains had an adenine. Two other SNPs occurred at positions 103 and 193 in some of the strains (Figure S3A). Ribosomal protein L32 gene (*rpmF*) (Figure S3B), as well as ribosomal proteins L35, L21, S18 and S16 showed that all CA clade strains differed from the HA clade strains by one to two nucleotides and there were no differences within a given clade. These results corroborated the molecular clock determination by SNP analysis. Using estimates of approximately 1% change every 25 million years in the 16S rRNA [24], [25], [26], a one to two nucleotide change out of 1569 bp (0.06–0.1%) would approximate the time of divergence of the two clades as somewhere between 1.5 million to 2.5 million years ago.

## Discussion

A number of previous publications have demonstrated differences between many hospital-associated and community-associated *E. faecium* strains, including differences in the rates of putative virulence genes, antibiotic resistance determinates, IS elements and transposons [1], [3], [5], [6], [8], [9], [10], [11], [13], [18]. Many of these, however, are part of the accessory genome presumably acquired through lateral gene transfer. Pyrosequencing and microarray studies also noted the genomic differences between such strains, suggesting the existence of two different clades, while still emphasizing the importance of the accessory genome differences in distinguishing these subpopulations [6], [18]. In a recent publication, we noted a large intra-species difference (3–10%) in the nucleotide sequences of *pbp5*, *pbp2*, *wlcA*, and *gls20* between HA clade and CA clade strains [19]. Interestingly, we subsequently noted an ∼6% difference (data not shown) between HA clade and CA clade strains in the *purK* allele (used for MLST) which also separated the strains into two distinct groups using UPGMA. Although not all HA-clade strains contained the *purK1* allele, we found that these strains were still distinctly different from strains in the CA clade (data not shown). With the advent of numerous draft genome *E. faecium* sequences and one closed *E. faecium* genome sequence (manuscript in preparation) and the fact that extensive analysis had yet to be reported regarding the core genomic differences, we sought to determine the extent of the differences between the two groups at a more fundamental level.

Consistent with our previous study of four genes [19] as well as consistent with the division seen in the phylogenomic tree for 7 of these strains using a concatenation of 649 proteins by van Schaik et al. [18], the difference in the concatenated sequences of 100 genes between the two clades is approximately 3.5–4.2%, clearly establishing the core genomic differences between these two subpopulations. The fact that >90% of the 100 core genes separated into two distinct groups and that the associated amino acid changes were found in most of the proteins analyzed and in a wide variety of metabolic and cellular processes (Table 3 and Table S1) , shows that there are likely differences between the two clades at a fundamental level. In addition, a relatively large number of the sequence changes between the strains (∼60%) were clade-specific changes. Changes in metabolism and cellular processes could be another reason why some strains adapt better to the hospital environment.

Not all strains that grouped genetically with the strains of the HA or CA clades had a hospital or community origin. One blood culture strain from a hospitalized patient, 1141733, always associated with strains in the CA clade, and E1039, a healthy volunteer fecal sample grouped with the HA clade. The fact that two of the 21 strains did not separate into the CA or HA clade according to origin demonstrates the complex ecology of colonizing and infecting *E. faecium*. A strain that did not fall strictly into one or the other clade was 1231408, which we call a hybrid strain (Table 2 and Table 3). In the SNP analysis, we were able to see that this strain recombined somewhere around ORF 10683, as the first part of its concatenated SNP sequence before ORF 10683 showed near identity with the SNP sequence of CA clade strains, and, after that point, its sequence showed near identity to the concatenated SNP sequence of the HA strains. Other evidence of recombination lies in the fact that, frequently, a few genes from a strain in one clade grouped with genes in the other clade a limited number per strain.

We also sought to estimate the time of separation of the HA and CA clades. A number of publications have tried to infer the molecular evolution of bacteria [20], [21], [23], [24], [25], [26], with two main strategies having been used: 16S rRNA and synonymous SNPs for the whole genome. Our 16S rRNA analysis showed a 0.06% to 0.1% difference between the two clades; this is a relatively large percentage within a species (Table 4 and Figure S3) and estimates the time of divergence between the clades as between 1.5–2.5 million years ago. However, since recent studies have expressed concerns about 16S rRNA being a reliable chronometer for bacterial evolution we also used the sSNPs to calculate the molecular clock. It has been suggested that the sSNP sites of protein-encoding genes reflect the underlying rate of mutation more reliably because they are not affected by selection or genetic drift and are distributed across genomes [22], [27]. This methodology seems to be especially useful for species with high levels of lateral gene transfer, such as *E. faecium* [22]. Whole genome SNP phylogenies have been shown to be highly accurate in terms of phylogeny and are more robust in defining deeper and higher resolution relationships among closely related individuals [22]. In a recent publication, it was determined that a select number of SNPs was sufficient to accurately determine the current phylogenetic position of any *B. anthracis* strain and could replace a tedious genome-wide SNP analysis, indicating that our approach, using 100 genes present in all strains and spread throughout the chromosome [27], was justified.

According to our sSNP molecular clock estimate using *E. coli* parameters, strains in the HA clade diverged from each other ∼100,000 to 300,000 years ago, whereas strains in the CA clade diverged from each other ∼300,000 to 900,000 years ago, corroborating the idea that HA clade strains in the available collection stem from a relatively recent ancestor. The sSNP analysis estimates, as well as the 16S rRNA analysis, the split between the two clades as somewhere around 1–3 million years ago. Even using the higher mutational rate used for *B. anthracis*, the estimated divergence time was 300,000 years ago. This highlights the fundamental core genomic differences between the two clades that could (in addition to the accessory genomic differences) be a reason why some strains adapt to the hospital environment and become opportunistic pathogens, while other strains do not.

The estimates of the time of divergence above presume that the rate of evolution of the HA and CA strains has remained constant over time. However, the transition to the pathogenic role may be associated with an increase in the mutation rate through selection of mutator strains. Thus, an alternative hypothesis to a gradual evolution of these clades from a common ancestor is that a well-adapted strain entered a new niche and then accumulated spontaneous mutations in genes, for example, mismatch repair genes, that then allowed the strain to go through one or more periods of rapid evolution. Of interest, the *mutS* gene was one of the 100 genes analyzed and it also showed prominent differences between the two clades, with 9 amino acid differences although their effect on function is not known. Nonetheless, although the estimate for the time of divergence is a very crude one (as it is hard to determine the divergence of a species without a fossil record), it suggests, even using mutation rates up to 1000 fold higher than estimated for *B. anthracis*, that the CA clade and HA clade isolates diverged long before the modern antibiotic era and tertiary care environment.

In summary, a number of studies have previously shown that *E. faecium* hospital-associated strains differ from many community/commensal strains [1], [3], [5], [6], [7], [8], [9], [10], [11], [19] and it has been postulated that the driving force behind the recent success of this opportunistic pathogen in hospitals was the gain of mobile genetic elements carrying antibiotic resistance determinants, virulence and/or fitness factors [6], [18]. In this paper, we have shown, using 100 core genes, that *E. faecium* strains belong to one of two subpopulations, or clades, that differ by ∼3.5–4.2% at the DNA level and that the estimated time of divergence between these two clades is at least 300,000 years ago, based on estimates for *B. anthracis* and/or *E. coli* rates of mutation and generation times in nature. Furthermore, the HA clade strains are more closely related to each other and diverged from each other more recently compared to the CA clade strains. These data further clarify the evolutionary history of hospital-associated *E. faecium* and show the extent of the differences between the two clades at the core genomic, protein, and synonymous SNP, providing evidence that acquired elements are not the only factors behind the recent success of this opportunistic organism and suggest that divergence between and within the clades took place many years ago.

## Materials and Methods

### Selection of 100 orthologs

Genome sequences of 21 *E. faecium* available from NCBI were studied (Table 1); TC6, also available, was not included here because it is a transconjugant of one of the other 21 genomes. To investigate whether there is a clear separation at the genome level into distinct groups, we selected 100 orthologs. These 100 orthologs were selected based on position (spread over different regions of the chromosome (manuscript in preparation)) and their presence in all strains as housekeeping genes or putative non-antigenic genes, including ribosomal proteins. Ortholog groups of *E. faecium* genomes were identified using OrthoMCL program using BLASTP E value of 1e-5 and default MCL inflation parameter of 1.5 with 80% sequence identity and 60% match length cutoffs. However, only those genes with the same size in base pairs were chosen.

### Comparative DNA sequence analysis

The 100 chosen orthologs nucleotide sequences were concatenated into one continuous sequence for each of the 21 *E. faecium* strains and a pair wise analysis using the Poisson correction method [28] on MEGA 4.0.2 software was performed [29], [30]. UPGMA phylogenetic trees [31] were constructed using the ClustalW alignment of the concatenated sequence [32], [33], [34]. The divergence score was calculated by taking the distance of the branch lengths between two strains, divided by the total distance (or sum of all the branch lengths) and multiplying by 100 [29]. In addition, each individual gene for the 21 genomes was also analyzed separately using ClustalW and UPGMA trees were generated using MEGA 4.0.2. software and analyzed to see which branch it segregated to (community or hospital clade) [29], [30], [32], [34]. If there were not two distinct branches, the gene was excluded from this analysis (8 of the 100 were excluded, leaving 92 genes).

### SNP analysis

To further investigate the differences among *E. faecium* strains, all SNP differences were extracted from the aligned 21 concatenated sequences and were concatenated into one continuous DNA sequence for each strain, compared to each other for nucleotide identity and divergence, and an UPGMA tree was constructed using the same methodology stated previously. We also calculated the number of SNPs that were strain specific (defined as found in only one strain) versus those found in two or more strains.

The number of non-synonymous changes were analyzed and it was determined in which genes they existed. Strain specific amino acid changes were excluded. We then identified clade-specific non-synonymous changes, defined as those changes that were present in at least two community-associated *E. faecium* strains but not in *E. faecium* strains within the HA clade, or present in 14 of the HA strains but not in the community strains.

### Molecular clock estimation

The molecular clock estimation requires four components, the number of synonymous SNPs, the number of potential SNP sites, the mutation rate, and the number of generations per year [20], [21], [23]. In order to estimate the rate of evolution of the two subpopulations, the number of synonymous SNPs (sSNPs) from the 100 gene analysis determined above was used. The potential sSNP sites were calculated by finding all three-base codons that could be used within the 100 genes and adding together all the sSNP sites from each codon. The median number of sSNPs between the strains within a clade was used to calculate the molecular clock for strains within a clade. Similarly, the median number of sSNPs for each clade was used to compare between the two clades. Since the synonymous mutation rate for *E. faecium* is unknown, a synonymous mutation rate of 1.4×10^−10^ mutations per base pair per generation based on mutation rates from *Escherichia coli* was used [20], [21]. The number of generations per year of *Enterococcus* species in the host or in the environment is also unknown, so a range of possible generation times 100, 200, and 300 was used, also based on *E. coli* [20], [21]. Estimates for a closer relative *Bacillus anthracis* are approximately 43 generations per year and its mutation rate is 5.2×10^−10^, and this higher mutation rate was also used [20], [21], [23]. The following equation was used to determine the time of divergence for each comparison: the number of sSNP/(the number of possible sSNP sites×mutation rate×the number of generations per year×2). The “2” in the denominator of the equation is used to account for the time of divergence of the two genomes, or the two groups compared [20], [23], [24].

### Analysis of the 16S rRNA and estimation of the time of divergence

The 16S rRNA gene was sequenced using the universal 16S rRNA primers B27F, 785F, 805R, and primers designed for outside the 16S rRNA gene for *E. faecium*, 16SEfmOS F1 5′-ATCGCAAGATTGTTCGAAC -3′, and 16S EfmOSR2 5′-CTTAGAAAGGAGGTGATCCAG -3′. The entire 16S rRNA of TX16 (DO) and TX1330 (manuscript in preparation), as representatives of the two clades, were resequenced. Sequences were extracted from all other strains using the NCBI sequence. Strains that had an incomplete 16S rRNA sequence (E1636, 1231502, Com12, Com15, 1230933, TX0133A, E1039, and TX82) were also resequenced. The sequences were aligned and compared using MEGA 4.0.2 software. The SNPs and percent nucleotide difference between the strains were determined, and the determination of time for divergence was based on a 1% change per 25 million years ago for 16S rRNA [26].

## Supporting Information

Figure S1 **UPGMA phylogenetic tree of the concatenated amino acid sequence (35,606 bp).** The results are based on a pair-wise analysis of the concatenated 100 protein amino acid sequence for each of the 21 *E. faecium* strains using the Poisson correction method in MEGA4.0.2 software. An UPGMA tree was constructed depicting the evolutionary distance between these sequences. The tree is drawn to scale with the branch lengths representing the evolutionary distances, the scale can be seen at the bottom of the tree.
(TIF)

Figure S2 **UPGMA phylogenetic tree of the concatenated SNP sequence (5,392 bp).** The results are based on a pair-wise analysis of the concatenated SNP nucleotide sequence for each of the 21 *E. faecium* strains using the Poisson correction method in MEGA4.0.2 software. An UPGMA tree was constructed depicting the evolutionary distance between these sequences. The tree is drawn to scale with the branch lengths representing the evolutionary distances, the scale can be seen at the bottom of the tree.
(TIF)

Figure S3 **The UPGMA phylogenetic trees representing the evolutionary relationship between the 16S rRNA and ribosomal protein L32 (** ***rpmF*** **) of 21** ***E. faecium*** **strains.** For all phylogenetic trees, the evolutionary distances were calculated using the Poisson correction method and UPMGA trees constructed using MEGA4.0.2 software. The trees are drawn to scale with the branch lengths representing the evolutionary distances, the scale of each tree can be seen at the bottom of each respective tree. A) An UPGMA tree representing the evolutionary relationship of the strains using the 16S rRNA nucleotide sequence for each of the 21 *E. faecium* strains. The table next to the tree indicates the changes in the 16S rRNA gene sequence at each nucleotide position for each branch of the tree. B) An UPGMA tree representing the evolutionary relationship of the strains using the *rpmF* nucleotide sequence for each of the 21 *E. faecium* strains. The table next to the tree indicates the changes in the *rpmF* sequence at each nucleotide position for each branch of the tree.
(TIF)

Table S1 **100 genes chosen for analysis in this study.** Listed are the 100 genes chosen for analysis in this study. These 100 orthologs in the 21 genomes were selected based on position and their presence in all strains as housekeeping genes or putative non-antigenic genes, including ribosomal proteins. Ortholog groups of *E. faecium* genomes were identified using OrthoMCL program using BLASTP E value of 1e-5 and default MCL inflation parameter of 1.5 with 80% sequence identity and 60% match length cutoffs. Only those genes with the same size in base pairs were chosen. In the table is the open reading frame number as is listed in the genome file on NCBI, the start and stop site represented by the nucleotide numbers, the size of the open reading frame in base pairs, the name of the gene if annotated, and the description of the annotated function of that gene. ^a^ Genes left out of the individual gene analysis (i.e. genes that did not show clade distinctions). ^b^ Genes that do not have non-synonomous changes in their encoded protein. ^c^ Genes that have non-synonomous changes in their encoded protein, but are not clade specific. ^d^ Refers to the nucleotide start and end sites on the DO chromosome (manuscript in preparation). ^e^ An empty cell indicates a KEGG number was not identified.
(DOCX)

Table S2 **Percent identity and divergence score matrix of the concatenated amino acid sequence.** Listed are the percent identity and divergence scores of the community clade, hospital clade, and hybrid strains using the amino acid sequence for the 100 concatenated genes. The 100 chosen orthologs amino acid sequences were concatenated into one continuous sequence for each of the 21 *E. faecium* strains and a pair wise analysis using the Poisson correction method on MEGA 4.0.2 software was performed. UPGMA phylogenetic trees were constructed using the ClustalW alignment of the concatenated sequence. The divergence score was calculated by taking the distance of the branch lengths between two strains, divided by the total distance (or sum of all the branch lengths) and multiplying by 100. ^a^ The numbers in italics (upper left) are the percent identity and divergence scores of the CA strains. ^b^ The numbers in bold are the percent identity and divergence scores of the hybrid strain. ^c^ The numbers in regular text (down right) are the percent identity and divergence scores of the HA strains.
(DOC)

Table S3 **Percent identity and divergence score matrix of the concatenated SNP sequence.** Listed are the percent identity and divergence scores of the community clade, hospital clade, and hybrid strains using the concatenated SNP sequence of the 100 concatenated genes. All SNPs were extracted from the aligned 21 concatenated sequences and were concatenated into one continuous DNA sequence for each strain, compared to each other for nucleotide identity and divergence using the same methodology previously stated (see Table S2). ^a^ The numbers in italics (upper left) are the percent identity and divergence scores of the CA strains. ^b^ The numbers in bold are the percent identity and divergence scores of the hybrid strain. ^c^ The numbers in regular text (down right) are the percent identity and divergence scores of the HA strains.
(DOC)
