# Supplementary figures and images for: Genomic and SNP Analyses Demonstrate a Distant Separation of the Hospital and Community-Associated Clades of Enterococcus faecium
Source: PLoS One. 2012 Jan 26;7(1):e30187. doi: 10.1371/journal.pone.0030187 (PMC3266884; doi:10.1371/journal.pone.0030187)

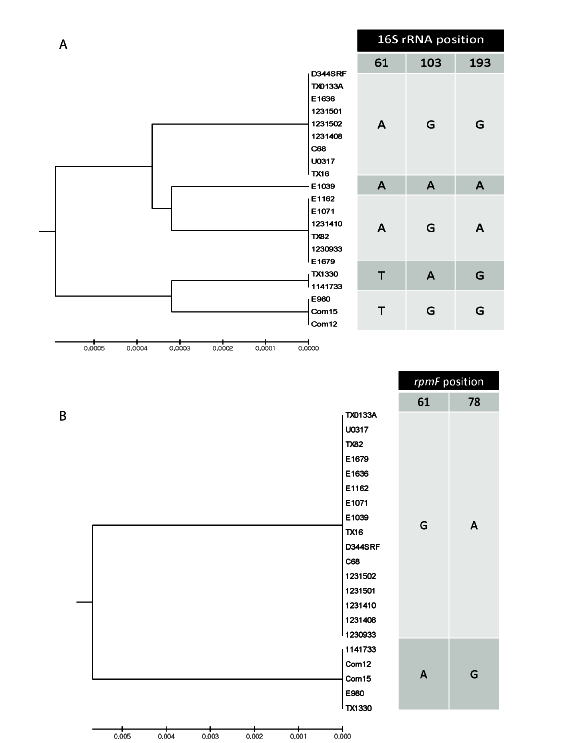

Supplement: Figure S3 — The UPGMA phylogenetic trees representing the evolutionary relationship between the 16S rRNA and ribosomal protein L32 ( rpmF ) of 21 E. faecium strains. For all phylogenetic trees, the evolutionary distances were calculated using the Poisson correction method and UPMGA trees constructed using MEGA4.0.2 software. The trees are drawn to scale with the branch lengths representing the evolutionary distances, the scale of each tree can be seen at the bottom of each respective tree. A) An UPGMA tree representing the evolutionary relationship of the strains using the 16S rRNA nucleotide sequence for each of the 21 E. faecium strains. The table next to the tree indicates the changes in the 16S rRNA gene sequence at each nucleotide position for each branch of the tree. B) An UPGMA tree representing the evolutionary relationship of the strains using the rpmF nucleotide sequence for each of the 21 E. faecium strains. The table next to the tree indicates the changes in the rpmF sequence at each nucleotide position for each branch of the tree. (TIF) [file pone.0030187.s003.tif]
