# Supplementary material for: Genomic and SNP Analyses Demonstrate a Distant Separation of the Hospital and Community-Associated Clades of Enterococcus faecium
Source: PLoS One. 2012 Jan 26;7(1):e30187. doi: 10.1371/journal.pone.0030187 (PMC3266884; doi:10.1371/journal.pone.0030187)
Supplement: Table S1 — 100 genes chosen for analysis in this study. Listed are the 100 genes chosen for analysis in this study. These 100 orthologs in the 21 genomes were selected based on position and their presence in all strains as housekeeping genes or putative non-antigenic genes, including ribosomal proteins. Ortholog groups of E. faecium genomes were identified using OrthoMCL program using BLASTP E value of 1e-5 and default MCL inflation parameter of 1.5 with 80% sequence identity and 60% match length cutoffs. Only those genes with the same size in base pairs were chosen. In the table is the open reading frame number as is listed in the genome file on NCBI, the start and stop site represented by the nucleotide numbers, the size of the open reading frame in base pairs, the name of the gene if annotated, and the description of the annotated function of that gene. a Genes left out of the individual gene analysis (i.e. genes that did not show clade distinctions). b Genes that do not have non-synonomous changes in their encoded protein. c Genes that have non-synonomous changes in their encoded protein, but are not clade specific. d Refers to the nucleotide start and end sites on the DO chromosome (manuscript in preparation). e An empty cell indicates a KEGG number was not identified. (DOCX) [file pone.0030187.s004.docx]

**Table S1.** 100 genes chosen for analysis in this study.

| **ORF** | **Start^d^** | **End** | **Strand** | **Size (bp)** | **Name** | **Description** |
| --- | --- | --- | --- | --- | --- | --- |
| HMPREF0351_10005^c^ | 4312 | 6258 | + | 1947 | *gyrB* | DNA topoisomerase subunit B |
| HMPREF0351_10009^b^ | 9872 | 10108 | + | 237 | *rpsR* | ribosomal protein S18 |
| HMPREF0351_10017 | 20752 | 22482 | - | 1731 | *poxB* | pyruvate oxidase |
| HMPREF0351_10054 | 58163 | 60250 | + | 2088 | *fusA* | protein-synthesizing GTPase |
| HMPREF0351_10068^a,b^ | 67123 | 67491 | + | 369 | *rplN* | ribosomal protein L14 |
| HMPREF0351_10078^b^ | 71325 | 72620 | + | 1296 | *secY* | preprotein translocase subunit SecY |
| HMPREF0351_10083^b^ | 74767 | 75705 | + | 939 | *rpoA* | RNA polymerase subunit alpha |
| HMPREF0351_10125^b^ | 123688 | 123909 | + | 222 | *metG* | methionine--tRNA ligase |
| HMPREF0351_10135 | 131070 | 133715 | + | 2646 | *mutS* | DNA mismatch repair protein MutS |
| HMPREF0351_10137 | 135863 | 136423 | + | 561 | *maf* | septum formation protein Maf |
| HMPREF0351_10164 | 162881 | 164251 | - | 1371 | *dinF* | MATE family protein |
| HMPREF0351_10231^b^ | 229498 | 229698 | + | 201 | *rpmI* | ribosomal protein L35 |
| HMPREF0351_10244^a,c^ | 240647 | 241726 | + | 1080 | *pepA* | M42 family glutamyl aminopeptidase |
| HMPREF0351_10375 | 354375 | 355688 | + | 1314 | *yieG* | NCS2 family purine:cation symporter |
| HMPREF0351_10389^c^ | 374025 | 374741 | + | 717 | *glpF2* | MIP family channel protein |
| HMPREF0351_10406 | 389976 | 390635 | + | 660 | *rcfA* | CRP/FNR transcriptional regulator |
| HMPREF0351_10439 | 429479 | 430207 | + | 729 |  | integral membrane protein |
| HMPREF0351_10460 | 453947 | 454675 | - | 729 | *agrA* | accessory gene regulator protein A |
| HMPREF0351_10474^b^ | 471998 | 472561 | + | 564 | *efp* | elongation factor P |
| HMPREF0351_10476 | 473048 | 474307 | + | 1260 | *lysA* | diaminopimelate decarboxylase |
| HMPREF0351_10493 | 491476 | 492381 | + | 906 |  | AraC family transcriptional regulator |
| HMPREF0351_10496 | 493430 | 494326 | + | 897 |  | LysM family surface protein |
| HMPREF0351_10540 | 536179 | 537375 | - | 1197 | *telA* | tellurite resistance protein |
| HMPREF0351_10568 | 567155 | 568342 | + | 1188 | *ftsW* | cell division membrane protein |
| HMPREF0351_10578^a^ | 575515 | 575985 | + | 471 | *iscU* | Fe-S cluster formation protein |
| HMPREF0351_10611 | 612012 | 612923 | + | 912 | *ppiA* | peptidylprolyl isomerase |
| HMPREF0351_10633 | 631143 | 631583 | + | 441 | *ntd* | nucleoside deoxyribosyltransferase |
| HMPREF0351_10659 | 664118 | 664564 | - | 447 | *nrdI* | ribonucleotide reductase |
| HMPREF0351_10683 | 684086 | 685021 | + | 936 | *murB* | N-acetylmuramate dehydrogenase |
| HMPREF0351_10719 | 723392 | 725002 | + | 1611 | *pyrG* | CTP synthase |
| HMPREF0351_10743^b^ | 745909 | 746256 | + | 348 | *yabA* | regulator of replication initiation |
| HMPREF0351_10765^b^ | 770338 | 770646 | + | 309 | *rplU* | ribosomal protein L21 |
| HMPREF0351_10791 | 791519 | 793711 | + | 2193 | *pbpA* | penicillin-binding protein 1 |
| HMPREF0351_10798 | 801185 | 801862 | + | 678 | *alr2* | alanine racemase |
| HMPREF0351_10801 | 803629 | 804336 | + | 708 | *divIVA* | cell division initiation protein DivIVA |
| HMPREF0351_10810 | 813420 | 816122 | + | 2703 | *sigL* | RNA polymerase sigma subunit |
| HMPREF0351_10903^a,c^ | 877677 | 878897 | + | 1221 | *tagH* | teichoic acid ABC superfamily |
| HMPREF0351_10938 | 914820 | 915452 | + | 633 |  | LuxR family response regulator |
| HMPREF0351_10945 | 920237 | 920845 | + | 609 | *sodA* | superoxide dismutase |
| HMPREF0351_10961^b^ | 937960 | 938226 | + | 267 | *ptsH* | PTS family porter component HPr |
| HMPREF0351_10976 | 950670 | 952049 | + | 1380 | *accC* | biotin carboxylase |
| HMPREF0351_11002 | 982334 | 984118 | + | 1785 | *pyk* | pyruvate kinase |
| HMPREF0351_11005^b^ | 985868 | 986047 | + | 180 | *rpmF* | ribosomal protein L32 |
| HMPREF0351_11081^c^ | 1066164 | 1067351 | + | 1188 | *tufA2* | elongation factor EF1A |
| HMPREF0351_11103 | 1091135 | 1091356 | - | 222 | *fer* | ferredoxin |
| HMPREF0351_11142 | 1127033 | 1127824 | + | 792 | *codY* | CodY family transcriptional regulator |
| HMPREF0351_11157 | 1143520 | 1144467 | + | 948 |  | LacI family transcriptional regulator |
| HMPREF0351_11204 | 1187269 | 1188564 | + | 1296 | *purB* | adenylosuccinate lyase |
| HMPREF0351_11221^a^ | 1205721 | 1207247 | + | 1527 | *araG* | L-arabinose ABC superfamily |
| HMPREF0351_11245 | 1232977 | 1233516 | + | 540 | *comEB* | competence protein ComEB |
| HMPREF0351_11282 | 1267078 | 1268121 | + | 1044 | *pheS2* | phenylalanine--tRNA ligase |
| HMPREF0351_11311 | 1296129 | 1298126 | + | 1998 | *tktA* | transketolase |
| HMPREF0351_11315^c^ | 1300562 | 1301008 | + | 447 | *fur2* | Fur family transcriptional regulator |
| HMPREF0351_11316 | 1301281 | 1302633 | + | 1353 | *nox* | NADH dehydrogenase |
| HMPREF0351_11335 | 1321937 | 1323286 | + | 1350 | *rhlB* | ATP-dependent RNA helicase |
| HMPREF0351_11369^a^ | 1356073 | 1356927 | + | 855 | *aroE* | shikimate dehydrogenase |
| HMPREF0351_11548 | 1517751 | 1520360 | - | 2610 | *clpB* | S14 family endopeptidase Clp |
| HMPREF0351_11556 | 1526794 | 1527720 | - | 927 | *dnaI* | primosomal protein DnaI |
| HMPREF0351_11568 | 1538758 | 1540938 | - | 2181 | *tex* | S1 domain RNA-binding protein |
| HMPREF0351_11610 | 1583663 | 1585222 | + | 1560 | *eriC* | CPA2 family cation:proton antiporter |
| HMPREF0351_11619^a,c^ | 1593185 | 1594474 | - | 1290 | *tig* | cell division trigger factor |
| HMPREF0351_11642 | 1618069 | 1618800 | - | 732 | *racX* | aspartate racemase |
| HMPREF0351_11679 | 1657393 | 1659102 | - | 1710 | *proS* | proline--tRNA ligase |
| HMPREF0351_11694 | 1673135 | 1674364 | - | 1230 | *arcA* | arginine deiminase |
| HMPREF0351_11718^b^ | 1693974 | 1695869 | - | 1896 | *asnB* | asparagine synthase |
| HMPREF0351_11738 | 1712076 | 1713422 | - | 1347 | *pgi* | glucose-6-phosphate isomerase |
| HMPREF0351_11759 | 1733232 | 1733942 | - | 711 | *alsD* | acetolactate decarboxylase |
| HMPREF0351_11836^b^ | 1805300 | 1805575 | - | 276 | *rpsP* | ribosomal protein S16 |
| HMPREF0351_11857^c^ | 1824675 | 1825649 | - | 975 | *cbh3* | choloylglycine hydrolase |
| HMPREF0351_11894 | 1855186 | 1856484 | - | 1299 | *asnS* | asparagine--tRNA ligase |
| HMPREF0351_11904^a^ | 1864309 | 1865616 | + | 1308 | *dacA* | S11 D-Ala-D-Ala carboxypeptidase |
| HMPREF0351_11979^c^ | 1928482 | 1929453 | - | 972 | *phoH* | phosphate starvation-inducible |
| HMPREF0351_12018^c^ | 1972203 | 1972880 | - | 678 | *phoU* | phosphate regulatory protein |
| HMPREF0351_12030 | 1984852 | 1985403 | - | 552 | *yfiA* | ribosome-associated inhibitor |
| HMPREF0351_12057 | 2005959 | 2006195 | - | 237 | *secG* | Sec family Type I secretory protein |
| HMPREF0351_12066 | 2013386 | 2014576 | - | 1191 | *pgk* | phosphoglycerate kinase |
| HMPREF0351_12075^c^ | 2021207 | 2021827 | - | 621 | *rpoE2* | RNA polymerase subunit delta |
| HMPREF0351_12120 | 2067231 | 2068871 | + | 1641 | *sfcA* | malate dehydrogenase |
| HMPREF0351_12172 | 2114033 | 2114770 | - | 738 | *glpQ2* | glycerophosphodiesterase |
| HMPREF0351_12186 | 2126586 | 2129231 | - | 2646 | *valS* | valine--tRNA ligase |
| HMPREF0351_12209 | 2155856 | 2156365 | + | 510 | *pgpA* | phosphatidylglycerophosphatase |
| HMPREF0351_12250 | 2197932 | 2199041 | + | 1110 | *msmK* | maltose ABC superfamily |
| HMPREF0351_12258^c^ | 2205177 | 2207186 | - | 2010 | *metG2* | methionine--tRNA ligase |
| HMPREF0351_12275 | 2222997 | 2224433 | - | 1437 | *bglB2* | beta-glucosidase |
| HMPREF0351_12305 | 2246582 | 2247934 | + | 1353 | *murE* | UDP- ligase |
| HMPREF0351_12332 | 2275213 | 2276637 | - | 1425 | *araA* | L-arabinose isomerase |
| HMPREF0351_12354^c^ | 2300216 | 2300500 | - | 285 | *groES* | chaperone GroES |
| HMPREF0351_12371 | 2316689 | 2317333 | - | 645 |  | GntR family transcriptional regulator |
| HMPREF0351_12412 | 2352668 | 2353534 | - | 867 | *rrmA* | 23S rRNA methyltransferase A |
| HMPREF0351_12423 | 2364403 | 2365056 | - | 654 | *mecA* | competence negative regulator MecA |
| HMPREF0351_12437 | 2384535 | 2385794 | - | 1260 | *cypX* | cytochrome P450 |
| HMPREF0351_12458 | 2401433 | 2402482 | - | 1050 | *comGB* | competence protein ComGB |
| HMPREF0351_12497 | 2452371 | 2453228 | - | 858 | *cscK3* | fructokinase |
| HMPREF0351_12540 | 2508096 | 2509289 | - | 1194 | *metK* | methionine adenosyltransferase |
| HMPREF0351_12545 | 2520662 | 2522773 | - | 2112 | *ftsH* | M41 family endopeptidase FtsH |
| HMPREF0351_12572 | 2552543 | 2553157 | - | 615 | *gmk* | guanylate kinase |
| HMPREF0351_12605^c^ | 2587423 | 2588580 | - | 1158 |  | PilT domain protein |
| HMPREF0351_12650 | 2625999 | 2626466 | - | 468 | *dps* | DNA-binding protein Dps |
| HMPREF0351_12682 | 2667041 | 2668387 | - | 1347 | *gor2* | glutathione-disulfide reductase |
| HMPREF0351_12694 | 2688720 | 2689610 | - | 891 | *spo0J* | stage 0 DNA-binding protein spoOJ |

^a^ Genes left out of the individual gene analysis (i.e. genes that did not show clade distinctions)

^b^ Genes that do not have non-synonomous changes in their encoded protein

^c^ Genes that have non-synonomous changes in their encoded protein, but are not clade specific

^d^ Refers to the nucleotide start and end sites on the DO chromosome (manuscript in preparation)

^e^ An empty cell indicates a KEGG number was not identified
