# Supplementary material for: Genomic and SNP Analyses Demonstrate a Distant Separation of the Hospital and Community-Associated Clades of Enterococcus faecium
Source: PLoS One. 2012 Jan 26;7(1):e30187. doi: 10.1371/journal.pone.0030187 (PMC3266884; doi:10.1371/journal.pone.0030187)
Supplement: Table S2 — Percent identity and divergence score matrix of the concatenated amino acid sequence. Listed are the percent identity and divergence scores of the community clade, hospital clade, and hybrid strains using the amino acid sequence for the 100 concatenated genes. The 100 chosen orthologs amino acid sequences were concatenated into one continuous sequence for each of the 21 E. faecium strains and a pair wise analysis using the Poisson correction method on MEGA 4.0.2 software was performed. UPGMA phylogenetic trees were constructed using the ClustalW alignment of the concatenated sequence. The divergence score was calculated by taking the distance of the branch lengths between two strains, divided by the total distance (or sum of all the branch lengths) and multiplying by 100. a The numbers in italics (upper left) are the percent identity and divergence scores of the CA strains. b The numbers in bold are the percent identity and divergence scores of the hybrid strain. c The numbers in regular text (down right) are the percent identity and divergence scores of the HA strains. (DOC) [file pone.0030187.s005.doc]

**Table S2.** Percent identity and divergence score matrix of the concatenated amino acid sequence.

| **Percent identity (%)** | | | | | | | | | | | | | | | | | | | | | | |
| --- | --- | --- | --- | --- | --- | --- | --- | --- | --- | --- | --- | --- | --- | --- | --- | --- | --- | --- | --- | --- | --- | --- |
| **Divergence** |  | **1141733** | **Com12** | **Com15** | **E980** | **TX1330** | **1231408** | **1230933** | **1231410** | **1231501** | **1231502** | **C68** | **D344** | **TX16** | **E1039** | **E1071** | **E1162** | **E1636** | **E1679** | **TX82** | **TX0133** | **U0317** |
|  | **1141733** |  | *99.8* | *99.6* | *99.5* | *99.8* | **99.1** | 98.7 | 98.7 | 98.8 | 98.7 | 98.7 | 98.7 | 98.7 | 98.8 | 98.7 | 98.7 | 98.7 | 98.7 | 98.7 | 98.7 | 98.7 |
|  | **Com12** | *0.2* |  | *99.5* | *99.6* | *100* | **99.1** | 98.7 | 98.7 | 98.8 | 98.7 | 98.7 | 98.7 | 98.7 | 98.8 | 98.7 | 98.7 | 98.8 | 98.7 | 98.7 | 98.7 | 98.8 |
|  | **Com15** | *0.4* | *0.5* |  | *99.6* | *99.5* | **99** | 98.7 | 98.7 | 98.8 | 98.7 | 98.7 | 98.7 | 98.7 | 98.8 | 98.7 | 98.7 | 98.7 | 98.7 | 98.7 | 98.7 | 98.7 |
|  | **E980** | *0.5* | *0.4* | *0.4* |  | *99.6* | **99** | 98.8 | 98.8 | 98.9 | 98.8 | 98.8 | 98.8 | 98.8 | 98.9 | 98.8 | 98.8 | 98.8 | 98.8 | 98.8 | 98.8 | 98.8 |
|  | **TX1330** | *0.2* | *0* | *0.5* | *0.4* |  | **99.1** | 98.7 | 98.7 | 98.8 | 98.7 | 98.7 | 98.7 | 98.7 | 98.8 | 98.7 | 98.7 | 98.8 | 98.7 | 98.7 | 98.7 | 98.8 |
|  | **1231408** | **0.9** | **0.9** | **1** | **1** | **0.9** |  | **99.5** | **99.5** | **99.3** | **99.5** | **99.5** | **99.4** | **99.5** | **99.3** | **99.5** | **99.5** | **99.3** | **99.4** | **99.5** | **99.5** | **99.5** |
|  | **1230933** | 1.3 | 1.3 | 1.3 | 1.2 | 1.3 | **0.5** |  | 100 | 99.7 | 99.9 | 99.9 | 99.8 | 99.9 | 99.7 | 99.9 | 99.9 | 99.7 | 99.8 | 99.9 | 99.9 | 99.9 |
|  | **1231410** | 1.3 | 1.3 | 1.3 | 1.2 | 1.3 | **0.5** | 0 |  | 99.7 | 99.9 | 99.9 | 99.8 | 99.9 | 99.7 | 99.9 | 100 | 99.7 | 99.8 | 100 | 99.9 | 99.9 |
|  | **1231501** | 1.2 | 1.2 | 1.2 | 1.1 | 1.2 | **0.7** | 0.3 | 0.3 |  | 99.7 | 99.7 | 99.6 | 99.7 | 99.7 | 99.6 | 99.7 | 99.7 | 99.6 | 99.7 | 99.7 | 99.7 |
|  | **1231502** | 1.3 | 1.3 | 1.3 | 1.2 | 1.3 | **0.5** | 0.1 | 0.1 | 0.3 |  | 99.9 | 99.8 | 99.9 | 99.7 | 99.9 | 99.9 | 99.7 | 99.8 | 99.9 | 99.9 | 100 |
|  | **C68** | 1.3 | 1.3 | 1.3 | 1.2 | 1.3 | **0.5** | 0.1 | 0.1 | 0.3 | 0.1 |  | 99.8 | 99.9 | 99.6 | 99.8 | 99.9 | 99.7 | 99.8 | 99.9 | 99.9 | 99.9 |
|  | **D344SRF** | 1.3 | 1.3 | 1.3 | 1.2 | 1.3 | **0.6** | 0.2 | 0.2 | 0.4 | 0.2 | 0.2 |  | 99.8 | 99.6 | 99.8 | 99.8 | 99.9 | 99.8 | 99.8 | 99.8 | 99.8 |
|  | **TX16** | 1.3 | 1.3 | 1.3 | 1.2 | 1.3 | **0.5** | 0.1 | 0.1 | 0.3 | 0.1 | 0.1 | 0.2 |  | 99.6 | 99.8 | 99.9 | 99.7 | 99.8 | 99.9 | 99.9 | 99.9 |
|  | **E1039** | 1.3 | 1.2 | 1.3 | 1.1 | 1.2 | **0.7** | 0.3 | 0.3 | 0.3 | 0.3 | 0.4 | 0.4 | 0.4 |  | 99.7 | 99.7 | 99.7 | 99.6 | 99.7 | 99.7 | 99.7 |
|  | **E1071** | 1.3 | 1.3 | 1.3 | 1.2 | 1.3 | **0.5** | 0.1 | 0.1 | 0.4 | 0.1 | 0.2 | 0.2 | 0.2 | 0.3 |  | 99.9 | 99.7 | 99.8 | 99.9 | 99.9 | 99.9 |
|  | **E1162** | 1.3 | 1.3 | 1.3 | 1.2 | 1.3 | **0.5** | 0.1 | 0 | 0.3 | 0.1 | 0.1 | 0.2 | 0.1 | 0.3 | 0.1 |  | 99.7 | 99.8 | 100 | 100 | 99.9 |
|  | **E1636** | 1.3 | 1.3 | 1.3 | 1.2 | 1.3 | **0.7** | 0.3 | 0.3 | 0.3 | 0.3 | 0.3 | 0.1 | 0.3 | 0.3 | 0.3 | 0.3 |  | 99.8 | 99.7 | 99.7 | 99.7 |
|  | **E1679** | 1.3 | 1.3 | 1.3 | 1.2 | 1.3 | **0.6** | 0.2 | 0.2 | 0.4 | 0.2 | 0.2 | 0.2 | 0.2 | 0.4 | 0.2 | 0.2 | 0.2 |  | 99.8 | 99.8 | 99.8 |
|  | **TX82** | 1.3 | 1.3 | 1.3 | 1.2 | 1.3 | **0.5** | 0.1 | 0 | 0.3 | 0.1 | 0.1 | 0.2 | 0.1 | 0.3 | 0.1 | 0 | 0.3 | 0.2 |  | 99.9 | 99.9 |
|  | **TX0133A** | 1.3 | 1.3 | 1.3 | 1.2 | 1.3 | **0.5** | 0.1 | 0.1 | 0.3 | 0.1 | 0.1 | 0.2 | 0.1 | 0.3 | 0.1 | 0 | 0.3 | 0.2 | 0.1 |  | 99.9 |
|  | **U0317** | 1.3 | 1.3 | 1.3 | 1.2 | 1.3 | **0.5** | 0.1 | 0.1 | 0.3 | 0 | 0.1 | 0.2 | 0.1 | 0.3 | 0.1 | 0.1 | 0.3 | 0.2 | 0.1 | 0.1 |  |

^a^ The numbers in italics (upper left) are the percent identity and divergence scores of the CA strains

^b^ The numbers in bold are the percent identity and divergence scores of the hybrid strain

^c^ The numbers in regular text (down right) are the percent identity and divergence scores of the HA strains
