# Supplementary material for: Genomic and SNP Analyses Demonstrate a Distant Separation of the Hospital and Community-Associated Clades of Enterococcus faecium
Source: PLoS One. 2012 Jan 26;7(1):e30187. doi: 10.1371/journal.pone.0030187 (PMC3266884; doi:10.1371/journal.pone.0030187)
Supplement: Table S3 — Percent identity and divergence score matrix of the concatenated SNP sequence. Listed are the percent identity and divergence scores of the community clade, hospital clade, and hybrid strains using the concatenated SNP sequence of the 100 concatenated genes. All SNPs were extracted from the aligned 21 concatenated sequences and were concatenated into one continuous DNA sequence for each strain, compared to each other for nucleotide identity and divergence using the same methodology previously stated (see Table S2). a The numbers in italics (upper left) are the percent identity and divergence scores of the CA strains. b The numbers in bold are the percent identity and divergence scores of the hybrid strain. c The numbers in regular text (down right) are the percent identity and divergence scores of the HA strains. (DOC) [file pone.0030187.s006.doc]

**Table S3.** Percent identity and divergence score matrix of the concatenated SNP sequence.

| **Percent identity (%)** | | | | | | | | | | | | | | | | | | | | | | |
| --- | --- | --- | --- | --- | --- | --- | --- | --- | --- | --- | --- | --- | --- | --- | --- | --- | --- | --- | --- | --- | --- | --- |
| **Divergence** |  | **1141733** | **Com12** | **Com15** | **E980** | **TX1330** | **1231408** | **1230933** | **1231410** | **1231501** | **1231502** | **C68** | **D344** | **TX16** | **E1039** | **E1071** | **E1162** | **E1636** | **E1679** | **TX82** | **TX0133** | **U0317** |
|  | **1141733** |  | *96* | *81* | *79* | *96* | **42** | 27 | 28 | 32 | 28 | 24 | 26 | 27 | 30 | 28 | 27 | 28 | 27 | 26 | 25 | 28 |
|  | **Com12** | *4* |  | *80* | *80* | *100* | **41** | 27 | 28 | 32 | 29 | 24 | 26 | 27 | 31 | 29 | 27 | 29 | 27 | 27 | 26 | 29 |
|  | **Com15** | *23* | *24* |  | *85* | *80* | **38** | 27 | 28 | 32 | 29 | 24 | 26 | 27 | 32 | 29 | 27 | 29 | 28 | 27 | 26 | 29 |
|  | **E980** | *26* | *24* | *17* |  | *80* | **39** | 32 | 33 | 37 | 34 | 29 | 31 | 32 | 36 | 33 | 31 | 34 | 32 | 31 | 31 | 34 |
|  | **TX1330** | *4* | *0* | *24* | *24* |  | **41** | 27 | 28 | 32 | 29 | 24 | 26 | 27 | 31 | 29 | 27 | 29 | 27 | 27 | 26 | 29 |
|  | **1231408** | **350** | **350** | **350** | **350** | **350** |  | **80** | **79** | **71** | **80** | **77** | **78** | **80** | **74** | **78** | **80** | **73** | **76** | **80** | **81** | **79** |
|  | **1230933** | 350 | 350 | 350 | 350 | 350 | **24** |  | 98 | 86 | 97 | 93 | 94 | 99 | 86 | 92 | 97 | 88 | 92 | 97 | 97 | 97 |
|  | **1231410** | 350 | 350 | 350 | 350 | 350 | **26** | 2 |  | 85 | 96 | 95 | 93 | 98 | 85 | 94 | 99 | 87 | 91 | 98 | 97 | 96 |
|  | **1231501** | 350 | 350 | 350 | 350 | 350 | **39** | 16 | 18 |  | 84 | 83 | 87 | 86 | 86 | 84 | 86 | 90 | 85 | 86 | 87 | 84 |
|  | **1231502** | 350 | 350 | 350 | 350 | 350 | **25** | 3 | 4 | 19 |  | 91 | 92 | 97 | 86 | 94 | 95 | 86 | 91 | 94 | 95 | 99 |
|  | **C68** | 350 | 350 | 350 | 350 | 350 | **28** | 8 | 5 | 20 | 10 |  | 91 | 93 | 82 | 91 | 96 | 85 | 89 | 96 | 94 | 90 |
|  | **D344SRF** | 350 | 350 | 350 | 350 | 350 | **27** | 7 | 8 | 15 | 9 | 10 |  | 94 | 86 | 91 | 94 | 94 | 95 | 94 | 96 | 91 |
|  | **TX16** | 350 | 350 | 350 | 350 | 350 | **25** | 1 | 2 | 16 | 3 | 7 | 6 |  | 86 | 92 | 97 | 88 | 92 | 97 | 97 | 97 |
|  | **E1039** | 350 | 350 | 350 | 350 | 350 | **35** | 16 | 17 | 16 | 16 | 21 | 16 | 16 |  | 86 | 85 | 91 | 85 | 85 | 86 | 86 |
|  | **E1071** | 350 | 350 | 350 | 350 | 350 | **28** | 8 | 7 | 19 | 6 | 10 | 10 | 8 | 16 |  | 95 | 87 | 92 | 95 | 93 | 95 |
|  | **E1162** | 350 | 350 | 350 | 350 | 350 | **24** | 4 | 2 | 16 | 6 | 4 | 6 | 3 | 17 | 5 |  | 89 | 92 | 100 | 98 | 94 |
|  | **E1636** | 350 | 350 | 350 | 350 | 350 | **36** | 13 | 14 | 12 | 15 | 17 | 6 | 13 | 10 | 14 | 12 |  | 92 | 89 | 90 | 86 |
|  | **E1679** | 350 | 350 | 350 | 350 | 350 | **30** | 8 | 10 | 17 | 10 | 13 | 5 | 9 | 17 | 9 | 8 | 9 |  | 92 | 94 | 91 |
|  | **TX82** | 350 | 350 | 350 | 350 | 350 | **24** | 4 | 2 | 16 | 6 | 4 | 6 | 3 | 17 | 5 | 0 | 13 | 9 |  | 98 | 94 |
|  | **TX0133A** | 350 | 350 | 350 | 350 | 350 | **23** | 3 | 4 | 15 | 5 | 6 | 5 | 3 | 16 | 7 | 2 | 11 | 7 | 2 |  | 95 |
|  | **U0317** | 350 | 350 | 350 | 350 | 350 | **25** | 3 | 5 | 19 | 1 | 10 | 9 | 4 | 16 | 6 | 6 | 15 | 10 | 6 | 6 |  |

^a^ The numbers in italics (upper left) are the percent identity and divergence scores of the CA strains

^b^ The numbers in bold are the percent identity and divergence scores of the hybrid strain

^c^ The numbers in regular text (down right) are the percent identity and divergence scores of the HA strains
